# Closing Editorial—Special Issue on “Vaccines and Vaccination: HIV, Hepatitis Viruses and HPV”

**DOI:** 10.3390/vaccines14050434

**Published:** 2026-05-12

**Authors:** Sonia Moretti, Ivan Schietroma

**Affiliations:** Italian National Institute of Health (ISS), Viale Regina Elena 299, 00161 Rome, Italy

We are pleased to present this closing editorial for the Special Issue “Vaccines and Vaccination: HIV, Hepatitis Viruses and HPV”, which we had the privilege of coordinating. This Special Issue brings together 13 contributions offering a timely overview of vaccine research, spanning global HPV (Human Papillomavirus) vaccination strategies, real-world determinants of vaccine uptake, immunogenicity in people living with HIV (Human Immunodeficiency Virus) and coinfections, as well as emerging evidence on therapeutic vaccine platforms.

The central role of HPV vaccination in cancer prevention has been consistently confirmed however, the articles also highlight how implementation barriers, financial constraints, and persistent inequalities continue to influence outcomes worldwide. At the same time, work focused on HIV and coinfections reminds us that immune restoration is often incomplete, and vaccine responses may remain suboptimal even among individuals with well-controlled infection.

Importantly, the studies addressing therapeutic vaccine approaches, together with economic evaluations targeting high-risk liver disease groups, reflect the expanding role of vaccines beyond prevention, toward immune support and, potentially, functional cure strategies.

We would like to sincerely thank all the authors who contributed to this Special Issue, as well as the *Vaccines* editorial team for their support throughout the process.

Branda et al. provide a comprehensive overview of HPV vaccination as a cornerstone of modern cancer prevention, emphasizing the disproportionate burden of HPV-related malignancies, particularly cervical cancer in low- and middle-income countries, and the growing relevance of HPV-associated oropharyngeal cancers. The authors discuss the virological and host genetic factors involved in carcinogenesis. While strong immunogenicity and the effectiveness of current prophylactic vaccines are supported, gaps in coverage linked to structural barriers and vaccine hesitancy persist. Advances in screening and therapeutic vaccine development are discussed [1].

Maness et al. examine behavioral interventions to improve HPV vaccine uptake among U.S. adolescents, identifying a lack of consistent equity-based analyses and limited assessment of differential effectiveness across demographic groups. Overall, the review highlights the need for standardized equity metrics and routine demographic stratification to ensure that vaccination strategies actively reduce existing disparities [2].

From an economic and policy standpoint, Mongan et al. synthesize cost-effectiveness evidence on HPV vaccination for cervical cancer prevention in the Asia–Pacific region, with particular relevance for Indonesia following the introduction of HPV vaccination into its national immunization program in 2023. The authors conclude that HPV vaccination remains a high-value intervention, particularly where screening infrastructure is weak, and they advocate for pricing-informed vaccine selection and strengthened delivery systems in Indonesia [3].

Shifting to real-world uptake in the post-pandemic setting, Scarbrough et al. report low HPV vaccination coverage among U.S. college students aged 18–26 years at a large public university in New York State. The results underline the ongoing need for targeted interventions in college-aged populations, particularly those centered on provider engagement and improved vaccine literacy [4].

Modi et al. confirm similar findings in HIV-positive women, showing that the lack of provider offer is a major barrier, while targeted counseling can increase acceptance. These findings support the integration of HPV vaccination counseling into routine HIV care as a practical strategy to reduce HPV-related morbidity and cervical cancer disparities in this vulnerable population [5].

Mooberry et al. review HPV vaccination in people living with HIV (PLWH), confirming its safety and immunogenicity but noting lower antibody titers and persistent implementation barriers. The review highlights the increased prevalence of HPV infection, persistence, and cancer risk among PLWH, particularly for cervical and anal cancers. The authors conclude that HPV vaccination should be embedded within HIV care pathways, alongside targeted education and health system interventions [6].

Evidence supporting an expanded role of HPV vaccination beyond primary prevention is presented by Erkmen and Arkan, who evaluate HPV clearance among HPV-positive women receiving the three-dose nonvalent vaccine series. These findings contribute to a growing body of evidence supporting HPV vaccination as a potential adjuvant strategy in secondary prevention and post-infection management [7].

Broadening the discussion to vaccination in immunocompromised populations, Muñoz-Gómez et al. assess COVID-19 vaccine responses in PLWH, showing generally adequate antibody development but weaker responses in those with advanced immunosuppression or incomplete viral control. These findings support the prioritization of PLWH in vaccination programs and provide a rationale for tailored booster strategies, particularly for those with low CD4+ counts [8].

Sepúlveda-Crespo et al. show that Hepatitis C virus (HCV) eradication does not restore HIV-specific humoral immunity in coinfected individuals. The findings suggest that HCV coinfection may leave a durable imprint on B-cell-mediated immunity, with implications for vaccine responsiveness and immune-based interventions even after successful cure [9].

Therapeutic vaccine development is addressed by Su et al., who evaluate a vesicular stomatitis virus (VSV)-based Hepatitis B virus (HBV) vaccine booster strategy supporting functional cure approaches. The findings highlight the importance of vaccine sequencing to overcome immune tolerance and support the potential of VSV-based boosting as part of functional cure strategies [10].

From a health economics perspective, Wang et al. demonstrate the cost-effectiveness of Hepatitis E virus (HEV) vaccination in Chinese adults with chronic liver disease. The analysis supports HEV vaccination as a high-value intervention in this high-risk population [11].

Returning to HBV control at the population level, Ango-Aguilar et al. provide a policy-focused review of HBV epidemiology in Peru, serving as a reminder that progress in viral hepatitis control is fragile and requires sustained investment in vaccination delivery, catch-up strategies, and targeted screening [12].

Finally, Amponsah-Dacosta et al. assess Hepatitis A virus (HAV) immunity among South African children with varying HIV exposure profiles. The findings support strengthened prevention measures, including water and sanitation interventions, public health education, and consideration of routine childhood HAV vaccination [13].

Taken together, the contributions in this Special Issue highlight the evolving scope of vaccinology, from population-wide prevention to targeted strategies for immunologically complex and high-risk groups.
